# An Improved Fast Prediction Method for Full-Space Bistatic Acoustic Scattering of Underwater Vehicles

**DOI:** 10.3390/s25082612

**Published:** 2025-04-20

**Authors:** Ruichong Gu, Zilong Peng, Yaqiang Xue, Cong Xu, Changxiong Chen

**Affiliations:** 1 School of Energy and Power Engineering, Jiangsu University of Science and Technology, Zhenjiang 212100, China; grc223@126.com (R.G.); yaqiangxue@just.edu.cn (Y.X.); xcong2025@163.com (C.X.); ccxsyyx@sina.com (C.C.); 2 Collaborative Innovation Center for Advanced Ship and Deep-Sea Exploration, State Key Laboratory of Ocean Engineering, Shanghai Jiao Tong University, Shanghai 200240, China

**Keywords:** bistatic scattering, monostatic scattering, sound field prediction, target strength, the monostatic to bistatic equivalence theorem

## Abstract

This paper presents an improved rapid prediction method for solving the full-space bistatic scattering sound field of underwater vehicles. The scattering sound field is represented as the product of the acoustic scattering transfer function and the sound source density function. By utilizing target surface mesh information and partial scattered sound pressure data as known inputs, the method predicts other bistatic scattering sound fields through numerical integration, matrix theory, and the least squares method. To reduce the data input required for predicting the scattering field, the monostatic to bistatic equivalence theorem is incorporated into the algorithm. A comparison with simulation results demonstrates that the proposed approach achieves favorable computational efficiency and reliability. Experimental tests on a double-layered ribbed cylindrical shell further validate the method’s performance.

## 1. Introduction

The underwater target acoustic scattering characteristics are important information sources for active sonar systems [1,2]. For surfaces with regular shapes, the solution to the Helmholtz equation can be expressed as a series expansion using the method of separation of variables. The series expansion is derived based on the type of incident wave and boundary conditions to obtain the numerical solution for the scattered wave. The surfaces that satisfy the separation of variables condition include a total of 11 target shapes, such as spheres [3,4], infinitely long cylinders [5], and others. Numerical methods include the T-matrix method [6], finite element/boundary element method (FEM/BEM) [7], and finite difference time domain (FDTD) method [8]. For high-frequency acoustic scattering calculations in large-scale models, numerical methods often face low computational efficiency due to the rapid increase in grid size. Therefore, physical acoustics methods, such as the Kirchhoff approximation method, are commonly used to solve high-frequency scattering problems in engineering. The planar elements method for sound field calculations proposed by Fan [9,10,11] et al. simplifies the Helmholtz–Kirchhoff integral formula to an area integral when the target size is much larger than the incident sound wavelength, and the target’s planar curvature is much larger than the wavelength. This approximation treats the incident wave as a plane wave. Sammelman [12] used smaller rectangular surfaces for approximate surface calculations but could not represent arbitrary-shaped curved surfaces. For targets with large surface curvature or complex shapes, Xue [13] employed the Gaussian–Legendre quadrature method with surface triangular elements for direct numerical computation of the area integral, offering faster computation speeds compared to traditional methods using planar triangular elements. When complex targets have concave surfaces or cavities, sound waves undergo multiple scatterings. Chai [14], based on the Kirchhoff approximation and ray-tracing theory, considered multiple scattering and shielding effects of sound waves and proposed a fast prediction method for calculating the scattered sound field of underwater corner-reflected composite targets. Wang [15] incorporated triangular meshing and iterative techniques into the numerical implementation, drawing on the iterative physical optics method [16] from electromagnetics, and proposed an iterative physical acoustics method (IPA) for fast computation of multiple scattering sound fields of concave targets. For simple convex smooth surface targets, saddle point or stationary phase methods [17] can be used to calculate the area integrals and obtain analytical expressions for the scattering fields of some regular-shaped targets, which also form the physical basis for the highlight model [18].

Multi-static sonar systems are commonly used for underwater target detection and identification due to their long operational range, good concealment, and resistance to interference. Many scholars have conducted in-depth research on multi-static acoustic scattering characteristics. Tan [19] proposed a sound transfer vector (ATV) method to calculate the bistatic target strength of underwater targets, but this method produces significant errors for irregular targets. Zheng [20] derived the Kirchhoff approximation formula for bistatic configurations with separated transmission and reception, combined with the planar elements method (PEM) for predicting underwater target echo characteristics, and established a bistatic sonar echo prediction model for small separation angles. Wang [21] proposed a Kirchhoff approximation method for large separation angles to calculate the bistatic acoustic scattering of rigid underwater targets. Liu [22] proposed a correction scheme for the scattering integral area of the target surface, thereby extending the applicability of physical acoustics to arbitrary bistatic angles. Schenck [23] expressed the scattering sound field as the product of the acoustic scattering transfer function and the equivalent sound source density function of the target surface, using known finite data to predict multi-static acoustic scattering for spherical targets. Chen [24,25] used numerical integration, matrix theory, and least squares methods to predict the bistatic scattering sound fields of spherical shells with panels and double-layer ribbed cylindrical shells, validating the effectiveness of the method through experiments. However, this method is not suitable for acoustic field prediction in non-centrosymmetric geometries and requires a large amount of input data.

To obtain the bistatic acoustic scattering sound field in conventional practice, it is necessary to calculate the monostatic and bistatic angles individually. However, in practical lake and sea trials, measuring the full-angle scattering sound field of a target presents significant challenges and high costs. Kell’s [26] Monostatic to Bistatic Equivalence Theorem (MBET) associates bistatic scattering with monostatic measurements through phase correction, which greatly reduces experimental costs. Bradley [27] further extended this theorem to the near-field region and explored the factors affecting its accuracy: simple scatterers (e.g., specular reflections) show good approximation when the bistatic angle is small, whereas multipath interactions or shadow effects amplify the errors. Gabig [28] noted that, for complex targets, the valid bistatic angle range is reduced by over 60% compared to simpler targets.

In this study, the transformation formulas are derived for the bistatic acoustic scattering characteristics transformation technique, which is applicable to rotationally symmetric models. Additionally, the introduction of the monostatic to bistatic equivalence theorem significantly reduces the input data required for predictions. The structure of this paper is as follows: In Section 2, the method for solving the scattering body’s acoustic scattering transfer matrix and sound source density function through numerical integration and the least squares method is derived, thereby obtaining the bistatic acoustic scattering sound pressure of the target. In Section 3, the applicability of the monostatic to bistatic equivalence theorem is first studied, and the scattering sound pressure at certain bistatic angles is replaced with the corresponding monostatic scattering sound pressure as input. The bistatic acoustic scattering acoustic field of the underwater vehicle is then predicted. In Section 4, experimental testing is conducted on a double-layer ribbed cylindrical shell. The conclusion is presented in Section 5.

## 2. Model Description

### 2.1. Multi-Static Acoustic Scattering Characteristics Transformation

**Figure 1:**
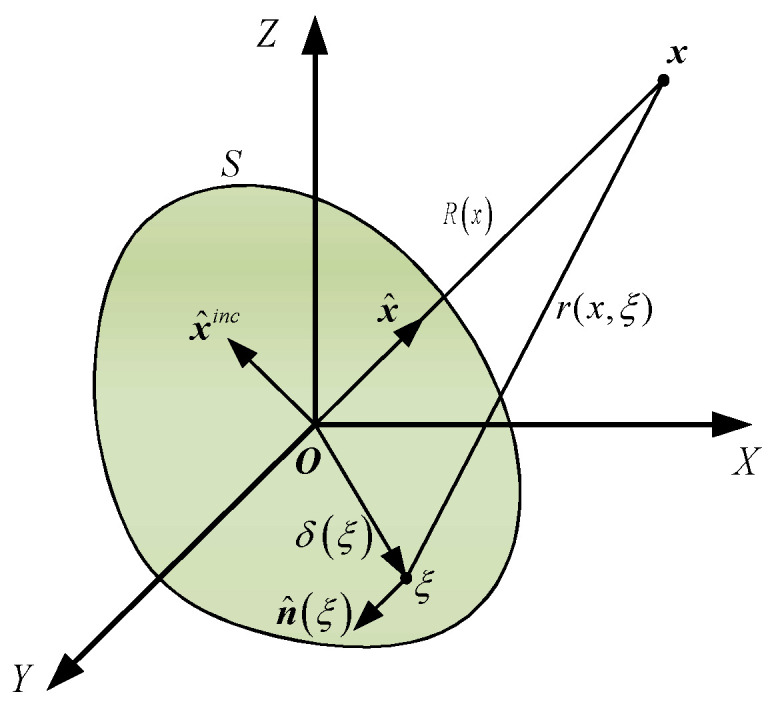
Schematic diagram of target acoustic scattering.

The schematic diagram of target acoustic scattering is shown in Figure 1. The scattered sound pressure of the target in the direction of the plane wave incidence can be expressed as the product of the acoustic scattering transfer function and the target surface sound source density function. According to reference [23], under the incidence of a plane wave with unit amplitude from direction ${\hat{x}}^{inc}$, the sound pressure at the receiving point x can be expressed as

(1) $$p^{S}(x,{\hat{x}}^{inc})=\frac{1}{4\pi }\int_{S}q(\xi ,{\hat{x}}^{inc})\left[\frac{\partial }{\partial n_{\xi }}+i\right]\frac{e^{-ikr\left(x,\xi \right)}}{r\left(x,\xi \right)}dS(\xi ),$$

where x is the receiving point; ${\hat{x}}^{inc}$ is the unit vector in the incident direction; S is the target surface; q represents the unknown sound source density function; ξ is any point on the target surface; r(x,ξ) is the distance between the target surface ξ and the receiving point x; R(x) is the distance between the origin O and the receiving point x.

The target surface S is discretized into $N_{S}$ surface elements. When the receiving point x is defined in the far-field of the integration surface S, r(x,ξ)≅R(x), and the far-field scattered sound pressure is defined as

(2) $$p_{ff}^{S}(\hat{x},{\hat{x}}^{inc})=\frac{1}{4\pi }\sum_{l=0}^{N_{S}}q_{l}({\hat{x}}^{inc})\times \int_{S_{l}}\left[ik\hat{x}\cdot \hat{n}(\xi )+i\right]\begin{array}{l} e^{-ik\hat{x}\cdot \delta (\xi )} \end{array}dS(\xi )$$

where $\hat{x}$ is the unit vector in the direction from the origin O to point x; $\hat{n}$ is the unit normal vector of the domain of point ξ; $\delta \left(\xi \right)$ is the vector from point ξ to the origin O.

Define matrix S, with its elements $S_{mn}=p_{ff}^{S}({\hat{x}}_{m},{\hat{x}}_{n}^{inc})$, and define matrix Q, with its elements $Q_{ln}=Q_{l}({\hat{x}}_{n}^{inc})$, resulting in

(3) $$S=C^{ff}Q$$

where the multi-static acoustic scattering matrix S is a $m_{1}\times n_{1}$-order matrix, where $m=1,2,\cdots ,m_{1}$, with m representing the receiving angle and $m_{1}$ representing the number of receiving angles. $n=1,2,\cdots ,n_{1}$, where n represents the incident angle and $n_{1}$ represents the number of incident angles. The acoustic scattering transfer matrix $C^{ff}$ is a $m_{1}\times l_{1}$-order matrix, where $l=1,2,\cdots ,l_{1}$, with l representing the element index and $l_{1}$ representing the total number of elements ($l_{1}=N_{S}$). The sound source density matrix Q is a $l_{1}\times n_{1}$-order matrix. The acoustic scattering transfer matrix $C_{ml}^{ff}$ is represented as

(4) $$C_{ml}^{ff}=\frac{1}{4\pi }\int_{S}\left[ik{\hat{x}}_{m}\cdot \hat{n}(\xi )+i\right]\begin{array}{l} e^{ik{\hat{x}}_{m}\cdot \delta (\xi )} \end{array}dS(\xi ).$$

#### 2.1.1. Calculation of the Sound Source Density Matrix

**Figure 2:**
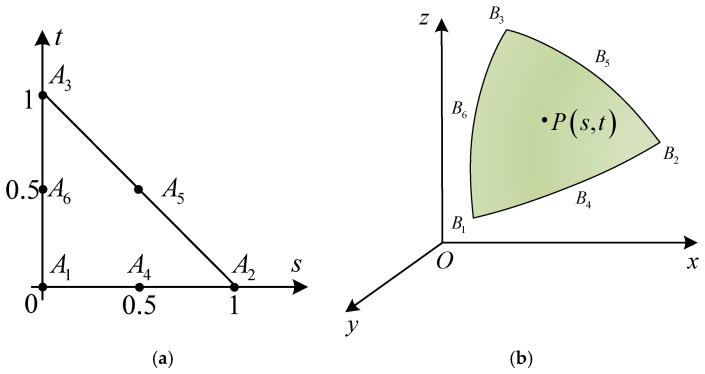
Mapping between the parametric triangle and the six-node triangle: (**a**) Parametric space triangle; (**b**) Physical space triangle.

The acoustic scattering transfer matrix $C_{ml}^{ff}$ is independent of the internal geometry of the object and only depends on the geometry of the object’s external surface. The target acoustic scattering transfer matrix $C_{ml}^{ff}$ for each surface element is computed using numerical integration methods [29], corresponding to the receiving angles within the calculation angle range, as described in Equation (4). First, the geometry of the target’s external surface is modeled, and a geometric mesh is generated using six-node triangular elements, with a total of $l_{1}$ elements. Figure 2 illustrates the relationship between the six-node triangle in physical space and the planar triangle in parameter space, along with their mapping.

The parametric element is defined by six nodes $A_{i}$ in the coordinates $\left(s,t\right)$. The points P on the curved surface triangle in Figure 2b can be expressed as

(5) $$P\left(s,t\right)=\sum_{i=1}^{6}R_{i}\left(s,t\right)B_{i},$$

where $B_{i}$ represents the vertices of the curved triangular element, and $R_{i}$ are the interpolation functions, defined as

(6) $$\left\{\begin{array}{l} R_{1}=(1-s-t)\left(1-2s-2t\right)\\ R_{2}=s\left(2s-1\right)\\ R_{3}=t\left(2t-1\right)\\ R_{4}=4s(1-s-t)\\ R_{5}=4st\\ R_{6}=4t(1-s-t) \end{array}\right.$$

In Equation (4), the integral can be expressed in the form of a Cartesian coordinate system f(x,y,z), transforming the surface integral concerning the acoustic scattering transfer matrix $C_{ml}^{ff}$ into a double integral, represented as

(7) $$C_{ml}^{ff}=\int_{S_{l}}f(x,y,z)dS_{l}=\iint_{D_{l}}f(x,y,z(x,y))\sqrt{1+{\left(\frac{\partial z}{\partial x}\right)}^{2}+{\left(\frac{\partial z}{\partial y}\right)}^{2}}dxdy$$

where the region $D_{l}$ represents the projection of the surface element $S_{l}$ onto the *xOy*-plane, and the partial derivatives of *z* with respect to *x* and *y* with respect to *s* and *t* are expressed as

(8) $$\left[\begin{array}{c} \frac{\partial z}{\partial x}\\ \frac{\partial z}{\partial y} \end{array}\right]=J^{-1}\left[\begin{array}{c} \frac{\partial z}{\partial S}\\ \frac{\partial z}{\partial t} \end{array}\right],J=\left[\begin{array}{cc} \frac{\partial x}{\partial S} & \frac{\partial y}{\partial S}\\ \frac{\partial x}{\partial t} & \frac{\partial y}{\partial t} \end{array}\right],$$

(9) $$\begin{array}{l} \frac{\partial z}{\partial S}=\sum_{i=1}^{6}\frac{\partial R_{i}}{\partial S}B_{i}=R_{i,S}B_{e}\\ \frac{\partial z}{\partial t}=\sum_{i=1}^{6}\frac{\partial R_{i}}{\partial t}B_{i}=R_{i,t}B_{e} \end{array},$$

where J represents the Jacobian matrix; $R_{i,s}$ and $R_{i,t}$ are the partial derivatives of the interpolation functions; $B_{e}$ refers to the coordinates of the six vertices of the surface triangular element. Using the Gauss–Legendre quadrature method [29], Equation (4) is expressed as

(10) $$C_{ml}^{ff}=\int_{0}^{1}\int_{0}^{1-s}f(s,t)\left|J\right|dsdt\approx \sum_{j=1}^{N}w_{j}f(\xi_{j},\eta_{j})\left|J\right|$$

where $\left(\xi_{j},\eta_{j}\right)$ represents the integration nodes; $w_{j}$ is the weight coefficient; N denotes the number of integration nodes.

#### 2.1.2. Calculation of the Sound Source Density Function

Rewrite the multi-static acoustic scattering matrix S and the sound source density function matrix Q in Equation (3) as column vectors $S_{ex}$ and $Q_{ex}$, respectively. The corresponding transformation of $C_{ml}^{ff}$ is $C_{ex}$, resulting in the system of equations

(11) $$S_{ex}=C_{ex}Q_{ex},$$

where the column vectors $S_{ex}$ and $Q_{ex}$ have $m_{1}n_{1}$ and $l_{1}n_{1}$ elements, respectively, and $C_{ex}$ is a $m_{1}n_{1}\times l_{1}n_{1}$-order matrix.

In general, $m_{1}=n_{1}$. According to acoustic reciprocity, the matrix S is symmetric. By eliminating the scattering sound pressure at the off-diagonal elements, we obtain a system of equations that only contains the diagonal elements of the acoustic scattering matrix. For the diagonal elements, the incident angle equals the receiving angle, which corresponds to the scattering sound pressure in a monostatic configuration. The resulting system of equations after elimination is entirely composed of monostatic scattering sound pressures, represented as

(12) $$S_{re}=C_{re}Q_{ex},$$

where the vector $S_{re}$ is composed of monostatic scattering sound pressures and the zero vector, expressed as $S_{re}=\left[S_{1},S_{2},S_{3}\right]$,where matrix $S_{1}$ is a row vector composed of monostatic scattering sound pressure elements; matrix $S_{2}$ is a zero vector obtained through the elimination based on the principle of acoustic reciprocity; matrix $S_{3}$ is a zero vector obtained through elimination based on the model symmetry. $C_{re}$ is the matrix transformation corresponding to the changes in the elements of $S_{re}$ to $Q_{re}$.

When the monostatic acoustic scattering matrix $S_{re}$ is known, i.e., when the monostatic scattering sound pressures are known, the acoustic source density matrix $Q_{ex}$ in Equation (12) can be solved using the least squares method. Since the number of equations in the system is greater than the number of unknowns (an overdetermined system), appropriately increasing the number of equations can improve the accuracy of the sound source density matrix. Several sets of bistatic scattering matrices $S_{ad}$ are added to the monostatic scattering matrix $S_{re}$. Meanwhile, the row vector corresponding to $S_{ad}$ is found in the acoustic scattering transfer matrix $C_{ex}$, forming the matrix $C_{ad}$, which is then appended to the matrix $C_{re}$, resulting in the mixed acoustic scattering matrix $S_{s}$ and the mixed acoustic scattering transfer matrix $C_{s}$. The expanded system of equations is as follows

(13) $$S_{s}=C_{s}Q_{ex},$$

where

(14) $$S_{s}=\left[\begin{array}{c} S_{re}\\ S_{ad} \end{array}\right],C_{s}=\left[\begin{array}{c} C_{re}\\ C_{ad} \end{array}\right].$$

The sound source density matrix $Q_{ex}$ is solved and transformed into a $l_{1}\times n_{1}$ matrix. This matrix, along with the computed acoustic scattering transfer matrix $C_{ml}^{ff}$ in Equation (4), is then substituted into Equation (3), leading to the calculation of the bistatic acoustic scattering sound pressure S.

### 2.2. Monostatic to Bistatic Equivalence Theorem

In sonar equations, target strength (TS) is commonly used to describe the strength of the target’s echo or its reflection ability. Target strength is defined as the decibel (dB) ratio of the sound intensity reflected back from the target at a distance of 1 m from its equivalent acoustic center to the sound intensity incident on the target from a distant source.

(15) $$TS=10\mathrm{lg}\frac{{\left.I_{S}\right|}_{r=1}}{I_{i}},$$

where $I_{i}$ is the sound intensity incident on the target; ${\left.I_{s}\right|}_{r=1}$ is the echo intensity at 1 m away from the target.

**Figure 3:**
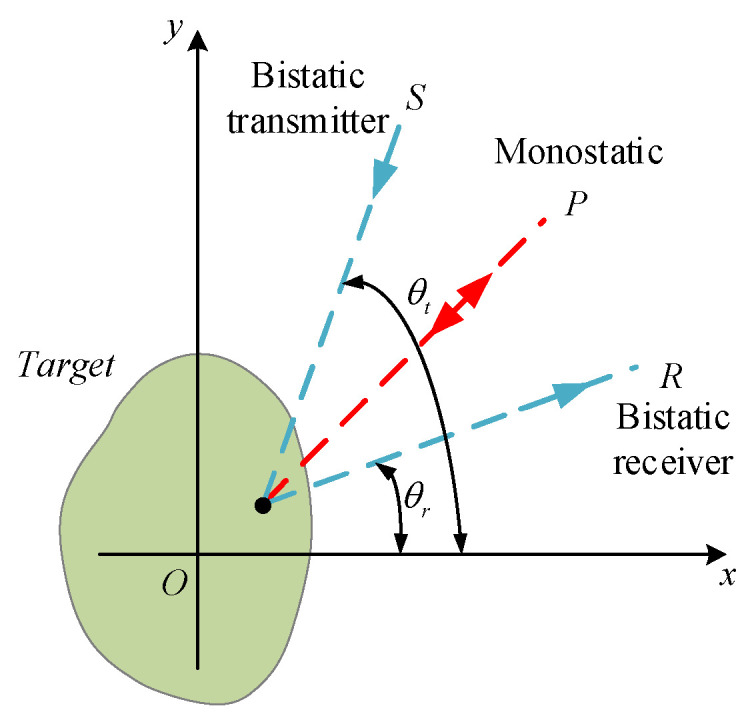
Schematic diagram of bistatic configuration.

Based on the physical optics method, for bistatic acoustic scattering at small bistatic angles in the far field, the bistatic target strength of a large, smooth object is equal to the monostatic target strength along the bisector between the bistatic angle of the source and receiver, as shown in Figure 3. The valid angular range for this assumption depends on the geometry of the object and is most applicable to scattering bodies dominated by specular reflection.

(16) $$TS_{b}(\theta_{t},\theta_{r},f)≅TS_{m}(\theta =\frac{\theta_{t}+\theta_{r}}{2},f),$$

where $TS_{b}$ and $TS_{m}$ represent the bistatic target strength and the equivalent monostatic target strength, respectively. $\theta_{t}$ and $\theta_{r}$ denote the bistatic incident angle and bistatic receiving angle, respectively. θ represents the equivalent monostatic angle, and f is the detection frequency.

## 3. Simulations

**Figure 4:**
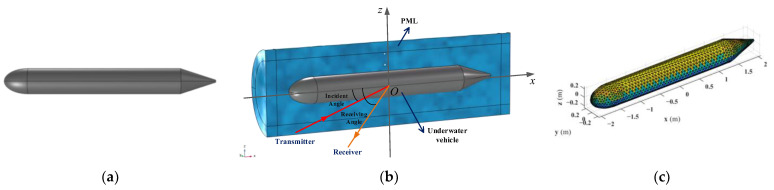
Schematic diagram of the underwater vehicle model: (**a**) Simplified geometric model; (**b**) Acoustic scattering calculation diagram; (**c**) The surface triangular mesh.

A simplified model of an underwater vehicle consists of a semi-ellipsoid, a cylinder, and a frustum (as shown in Figure 4a), with a total length of 4.13 m. The geometric parameters are provided in Table 1.

The bistatic acoustic scattering sound field of an underwater vehicle is analyzed using the 3D “Pressure Acoustics” module in COMSOL Multiphysics 6.1. The computational model is established as shown in Figure 4b, with the origin of the coordinate system located at the geometric center of the vehicle. The outermost layer of the model is a perfectly matched layer (PML), simulating an infinitely extended domain with non-reflective, absorbing boundaries. The external medium surrounding the underwater vehicle is water, while the interior is filled with air.

A plane wave with a pressure of 1 Pa is added along the *xOy* plane in the external water region through the “Background Pressure Field” module. The incident angle is defined as 0° along the positive *x*-axis. The Helmholtz–Kirchhoff integral is solved using the “External Field Calculation” module, which employs the full integral method. Based on the acoustic impedance boundary conditions between water and air, the “Frequency Domain” study is performed with both the incident and receiver angles ranging from 0° to 360°, with increments of 2°. The frequency range for the analysis spans from 100 Hz to 1 kHz, with a frequency step size of 50 Hz.

The results of this analysis are used to calculate the scattering sound field at a receiver located 10 km from the geometric center of the model in the far-field region. This is achieved using the “Global Computation” module, which provides the scattered sound field at the receiver, enabling further analysis of the wave propagation and scattering characteristics in the far-field zone. During the computation, the maximum element edge length of the mesh is constrained to one-sixth of the wavelength corresponding to the highest frequency. To predict the multi-static sound field, the surface triangular mesh used to compute the scattering transfer function ($C^{ff}$) is shown in Figure 4c, consisting of 814 mesh elements.

**Figure 5:**
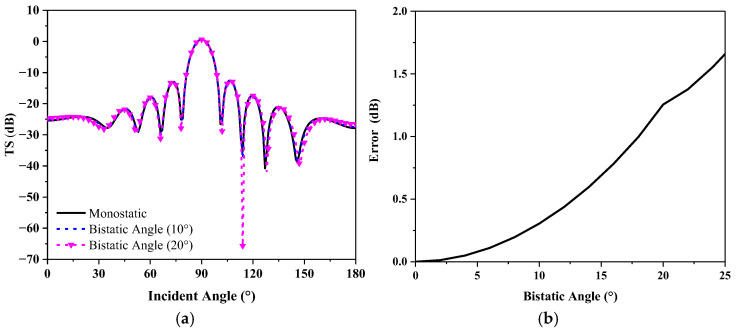
Target strength of the underwater vehicle at 1000 Hz: (**a**) Directivity; (**b**) Mean error.

**Figure 6:**
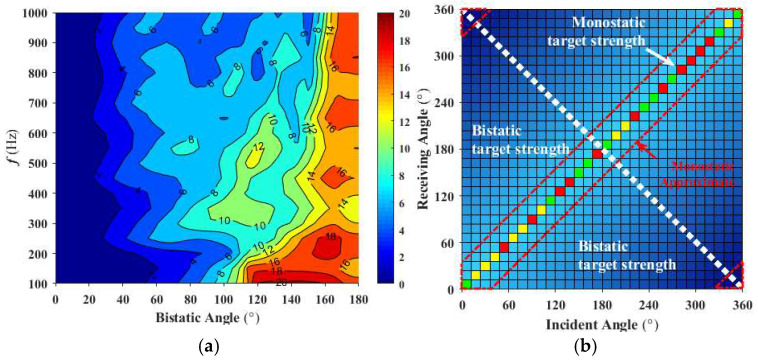
Monostatic to bistatic equivalence theorem calculation: (**a**) Mean error from 100 Hz to 1 kHz; (**b**) Applicable data distribution range.

Based on finite element method (FEM) simulation data, the target strength along the bisector of the central angle at 1 kHz for bistatic angles of 10° and 20° was calculated, along with the horizontal directivity and error at the actual monostatic angle, as shown in Figure 5. The error in target strength between the equivalent monostatic angle and the actual monostatic angle along the bisector of the bistatic angle, for bistatic angles ranging from 0° to 180° between 100 Hz and 1 kHz, is shown in Figure 6a. The data distribution range is shown in Figure 6b, with different colored sections along the diagonal representing the actual monostatic angle, and the red box highlighting the data range that can be equivalently treated as bistatic angles. It can be observed that, for bistatic angles within 20°, when the mean error is 1.3 dB, the target strength along the bisector of the bistatic angle can be approximately equivalent to the target strength at the monostatic angle. 

**Figure 7:**
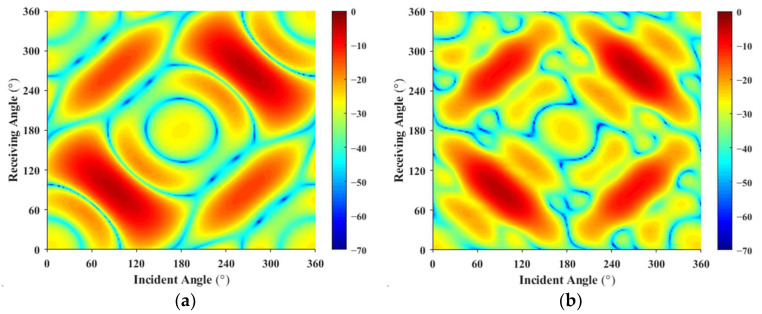
Scattered sound pressure at the incident-receiving angle at 500 Hz: (**a**) Actual; (**b**) Reconstructed.

The multi-static acoustic scattering data for each frequency are processed, and based on the monostatic to bistatic equivalence theorem, the angles along the bisector of the bistatic angle range from 0° to 20° are replaced with the corresponding monostatic scattering sound pressure. This approach selects only the monostatic angle data to construct the multi-static acoustic scattering matrix S as the forecast input. At 500 Hz, the reconstructed target incident-receiving angle scattering sound pressure is shown in Figure 7. Due to the complexity of the computational model, using only monostatic scattering sound pressure data to estimate the source density function via the least squares method results in a large deviation, with a mean error of 6.01 dB.

Based on the known monostatic target strength data from 0° to 358°, additional bistatic data are added as forecast input for the acoustic scattering matrix.

Condition 1: Bistatic data along the diagonal above (Figure 6b) are selected at 12° intervals (i.e., for a 0° incident angle, the receiver angle ranges from 12° to 348° with a 12° interval; for a 12° incident angle, the receiver angle ranges from 24° to 348° with a 12° interval; and so on, until a 336° incident angle with a receiver angle of 348°). At this point, the ratio of input elements to forecasted elements is 1.935%.

Condition 2: According to the monostatic to bistatic equivalence theorem, the bistatic scattering pressure data at a 12° bistatic angle from Condition 1 are replaced with corresponding monostatic scattering pressure data (i.e., the scattering pressure at a 0° incident angle and 12° receiver angle is replaced with the monostatic scattering pressure at a 6° angle, at a 12° incident angle and 24° receiver angle it is replaced with a monostatic scattering pressure at an 18° angle, and so on, until the data at a 336° incident angle and 348° receiver angle are replaced with the monostatic scattering pressure at a 342° angle). In this case, the ratio of input elements to forecasted elements is 1.842%.

**Figure 8:**
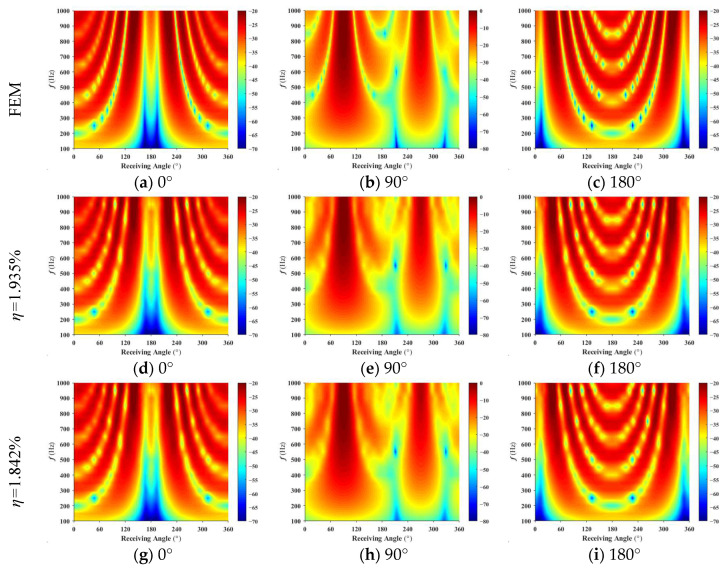
Receiving angle-frequency spectrum: (**a**–**c**) represent the finite element method results; (**d**–**i**) show the forecast results for 1.935% and 1.842%, respectively.

**Figure 9:**
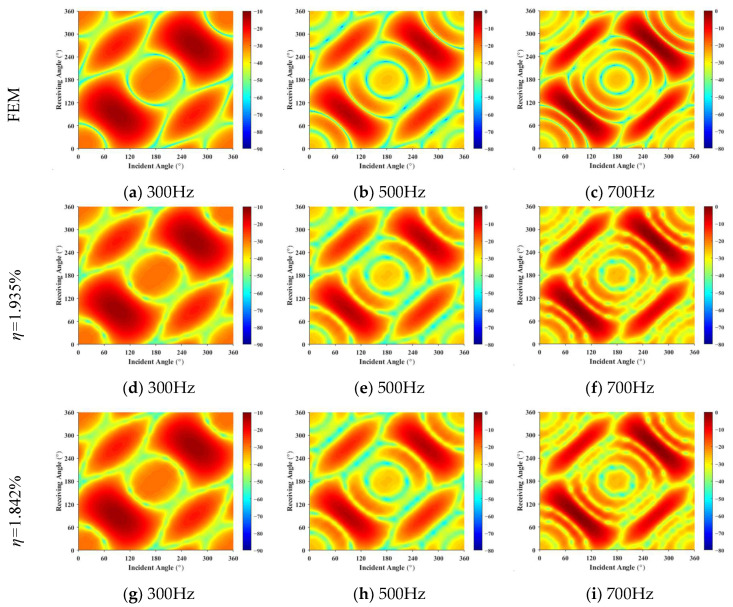
Incident-receiving angle spectrum: (**a**–**c**) represent the finite element method results; (**d**–**i**) show the forecast results for 1.935% and 1.842%, respectively.

**Figure 10:**
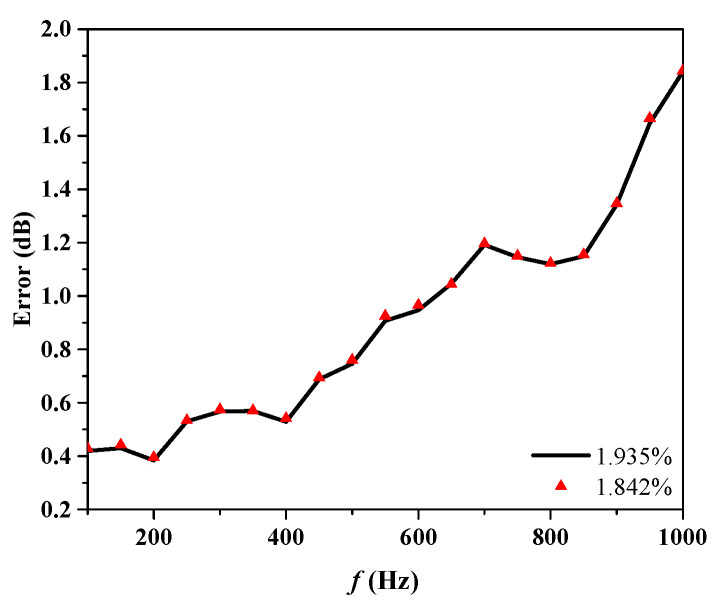
The mean errors of Condition 1 and Condition 2.

The scattering pressures from Condition 1 and Condition 2 are used as the forecast input acoustic scattering matrix. Using the least squares method, the reconstructed receiving angle-frequency spectra and incident-receiving angle spectra are shown in Figure 8 and Figure 9. The error curves for both methods, as they vary with frequency, are shown in Figure 10.

Based on Figure 8, Figure 9 and Figure 10, the accuracy of target strength prediction is significantly improved by incorporating bistatic data alongside the known monostatic data. Under two different conditions, where the ratio of input elements to forecasted elements is 1.935% and 1.842%, the target strength predicted using the least squares method shows a high degree of agreement with the finite element calculation results. As shown in Figure 10, after applying the monostatic to bistatic equivalence theorem, the scattering pressure data required for prediction can be reduced by 4.715%, while the mean error remains nearly the same as before the equivalence.

As the frequency increases, the mean error in the predicted target intensity tends to increase, necessitating the use of more bistatic data to achieve higher prediction accuracy. At lower frequencies, the source density function is relatively simple, and a combination of monostatic data along with a limited amount of bistatic data is sufficient for accurate estimation. However, as the frequency rises, the source density function becomes increasingly complex, requiring a larger amount of bistatic data to accurately estimate it.

## 4. Experimental Results

**Figure 11:**
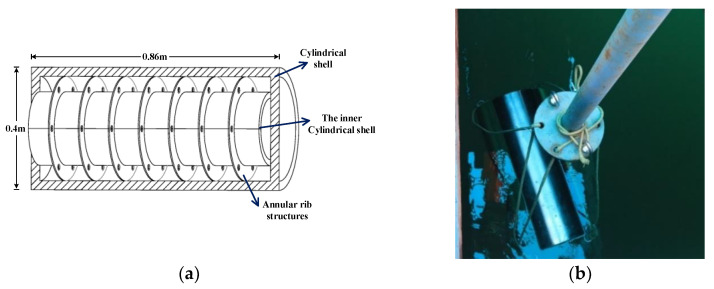
Schematic diagram of the double-layered ribbed cylindrical shell for the experiment: (**a**) Cross-section drawn; (**b**) Photograph of the model launched.

**Figure 12:**
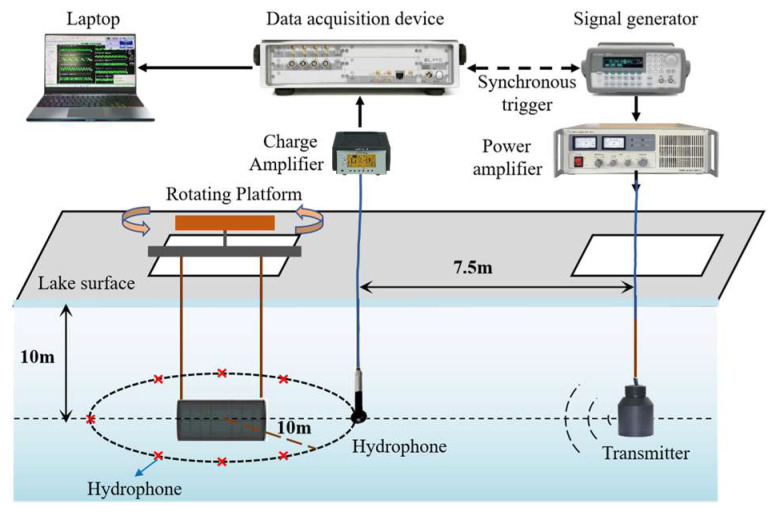
The experimental setup.

The experiment is conducted on a double-layered ribbed cylindrical shell to validate the feasibility of the bistatic acoustic scattering sound field prediction method. The test model is made of carbon steel (as shown in Figure 11), with a total length of 0.86 m and a radius of 0.2 m. The outer shell is a cylindrical shell with a thickness of 30 mm, while the inner cylindrical shell has a length of 0.8 m and a radius of 0.1 m. Seven 4 mm-thick annular rib structures are evenly distributed between the inner and outer shells, each with six circular holes, each having a radius of 10 mm. The interior of the shell is filled with air. The experimental setup is shown in Figure 12, where the transmitter, hydrophones, and model are all positioned at the same depth. The transmitter emits frequencies ranging from 500 Hz to 1 kHz, and eight hydrophones are distributed at 45° intervals along the geometric center of the model. The hydrophone located along the center of the cylindrical shell, at the position aligned with the transmitter, is defined as 0° (near the transmitter). In the experiment, the sound source position is kept fixed, while the flange is rotated to allow the model to rotate horizontally at a constant speed over a full 360°.

**Figure 13:**
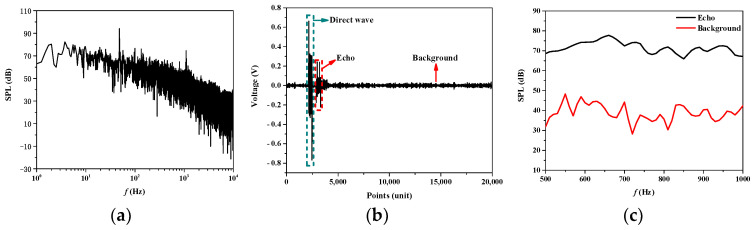
Comparison of echo signal and background noise: (**a**) Background noise of Qiandao Lake; (**b**) Time-domain signal; (**c**) Echo-to-noise ratio.

The background noise measured during the experiment is shown in Figure 13a. The background noise level ranges from approximately 30 dB to 70 dB between 500 Hz and 1000 Hz, meeting the environmental requirements for low background noise necessary for the experiment. After processing the experimental data, the time-domain signal measured by the hydrophone at a 0° incident angle and a 0° reception angle at 1 kHz is shown in Figure 13b. The hydrophone sequentially receives the direct wave, echo, and background noise signals. The echo-to-noise ratio, as shown in Figure 13c, exceeds 20 dB, confirming that the echo signal is valid.

**Figure 14:**
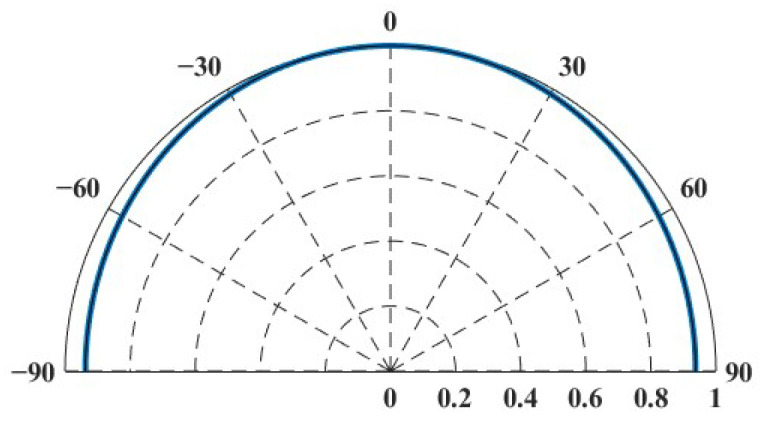
Spatial directivity of the transducer at 1 kHz.

**Figure 15:**
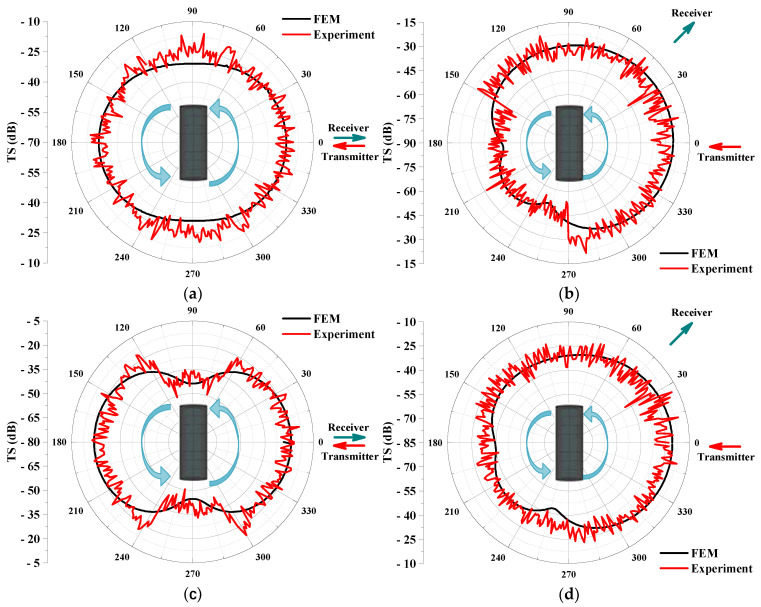
Comparison of experiment results and FEM: Target strength directivity of the 0° hydrophone at 500 Hz (**a**) and 700 Hz (**c**); Target strength directivity of the 45° hydrophone at 500 Hz (**b**) and 700 Hz (**d**).

Due to the long wavelength of low-frequency sound waves, they are prone to diffraction during propagation, resulting in a relatively uniform sound intensity within the propagation range, as shown in Figure 14. This makes it difficult to achieve significant focusing effects in any particular direction. Therefore, the sound source level and directivity of the transducer were initially calibrated before the experiment. Multiple repetitive measurements were carried out at 0° and 45° azimuths, and the measured signals were averaged to mitigate measurement errors caused by inhomogeneous transducer directivity. At the same time, the signals were band-pass filtered in the frequency range of 300 Hz to 1500 Hz in order to reduce the effect of background noise. The target strength directivity obtained from the experimental data is shown in Figure 15. The experimental results generally exhibit good agreement with the finite element method (FEM) results.

**Figure 16:**
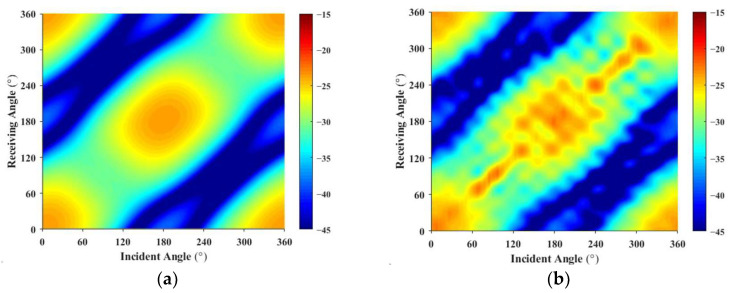

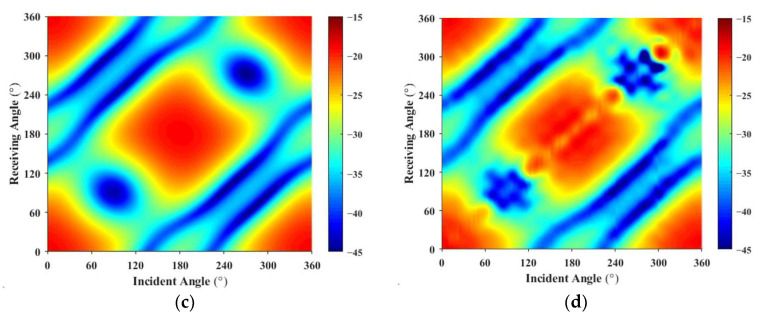
Multi-static target strength map: Comparison of FEM (**a**) and experimental predictions (**b**) at 500 Hz; Comparison of FEM (**c**) and experimental predictions (**d**) at 700 Hz.

By inputting all the monostatic transceiver data and the bistatic data from seven receiver angles, the ratio of input data to predicted data is 4.4%. The predicted bistatic acoustic scattering sound field for the double-layer ribbed cylindrical shell at 500 Hz and 700 Hz is shown in Figure 16. The forecasted results based on the experimental data exhibit characteristics similar to those from the finite element method (FEM) calculations, with mean errors in target strength of 3.5 dB and 5.8 dB, respectively, indicating a significant discrepancy. It is evident that, since the sound source density function is predicted from the input acoustic scattering pressure data, large errors in the input data lead to substantial inaccuracies in estimating the sound source density function.

## 5. Conclusions

This paper presents an improved method for predicting the bistatic acoustic scattering sound field of underwater vehicles. The method forecasts the bistatic acoustic scattering sound field by inputting target grid information and a small amount of bistatic scattering sound pressure along with monostatic data. By applying the monostatic to bistatic equivalence theorem, scattering sound pressures at some receiver–transmitter angles are replaced by those at the corresponding monostatic angles, thus reducing computational and testing costs. The results show that the method effectively predicts the bistatic acoustic scattering sound field, and the following conclusions can be drawn:

(1) This method extends the prediction technique to the scattering sound field of rotating targets in underwater vehicles. It requires only the target surface grid and a small amount of known scattering sound pressure data. When the ratio of input data to forecast data is 1.935%, the scattering characteristics of the vehicle are predicted with a reasonable degree of accuracy from 100 Hz to 1 kHz.
(2) The introduction of the monostatic to bistatic equivalence theorem into the scattering sound field prediction method reduced the input data by 4.7%, while maintaining similar prediction accuracy, significantly reducing the workload for testing or computation.
(3) Experimental data measured at 500 Hz and 700 Hz were used to further validate the effectiveness of the proposed prediction method. When the ratio of input experimental data to forecast data was 4.4%, the average errors were 3.5 dB and 5.8 dB, respectively.

## Figures and Tables

**Table 1:** Dimensions of underwater vehicle components.

Position	Component Parts	Geometric Parameters (Unit: m)	
Bow	semi-ellipsoid	Long Semi-Axis	0.46
		Short Semi-Axis	0.25
Midship	Cylinder	Radius	0.25
		Height	2.97
Stern	Frustum	Large Bottom Radius	0.25
		Small Bottom Radius	0.02
		Height	0.70

## Data Availability

All evaluated data are presented in this paper in graphical form. Original measurement data of this study are available upon request from the corresponding author.
